# The Hering Memorial Volume

**Published:** 1881-08

**Authors:** E. W. Berridge

**Affiliations:** London


### THE MEMORIAL VOLUME.

E. W. Berridge, M. D., London.

An appeal has been made to each member of our profession, through our journals, to subscribe what sums he thinks fit for the publication of a Memorial Volume to our beloved colleague, the late Constantine Hering: the said volume to consist of a short sketch of his career, followed by the eulogies pronounced upon him after his departure from us, and all surplus money to be handed to Mrs. Hering. I am informed, however, that only about $600 have been subscribed, less than the anticipated cost of the volume itself, and that the third volume of the *Guiding Symptoms*, which was in press as far as *Cainca* last June, when I was in Philadelphia, has been stopped from want of funds.

It appears to me, that while the intention of the promoters of this memorial scheme was excellent, their plans are open to much criticism. Their object seems to have been two-fold: (1.) to honor the memory of the departed; and (2.) to help his widow, to whom he was able to leave but little.

Now, as to the first of these intentions, I should delight to read, not a short sketch merely, but a full and exhaustive account of Dr. Hering's life; but I do not care to read a large volume of what others have said about him, especially seeing that many of his eulogizers are mongrels who now desire to affiliate themselves to his great name, so as to bolster up their own ignorance and falsehood.* The best memorial to Hering, will be to publish what he has written himself, not what others have written about him. With regard to the second intention, to provide a fund for Mrs. Hering, it is a thoroughly just and proper scheme, and one to which I shall be most happy to contribute; but I do not care to do so if the only, or even the chief, result is merely the publication of the Memorial Volume, which seems at present to be most likely.

Why should we not carry out Dr. Hering's own desires? He told me last June, that he depended upon the sale of his *Guiding Symptoms* to bring in eventually an income for his wife, for he had but little to leave her. Let us now do the work in the old hero's way. Let us each purchase a copy of the *Guiding Symptoms*—those who are wealthy can purchase two—and let us all urge our patients, as well as our colleagues, to imitate our example. I have myself secured *seven* professional subscribers to the work, and *five* among my own patients, and I hope to do still more in this direction; so there is no lack of encouragement. And, if possible, along with the dedication to Dr. P. P. Wells, which Hering told him in my presence he intended to prefix to the third volume (Hering considering that its completion would then be assured), let us have a full account of his life with a portrait.

---

### FLUXION DILUTIONS.

Eldridge C. Price, M. D., Baltimore, Md.

The ability to perfectly express an idea is a gift. The ability to perfectly understand an idea is also a gift. Few men possess either, fewer possess both.

---

*"*Hypocrisy* here comes in and plays its dreadful part. It is neither reputable nor profitable to stand with an exponent of the highest discoverable good while he lives, *but after his decease the world fastens on his memory*, with a blind respect that sometimes becomes a more blind adoration. *So the hypocrites make a commodity of this regard and worship*, and by affecting to be followers of this departed virtue, exponents of its principles, and inheritors of its inspiration, *they build themselves in power and reputation*."—Thomas Lake Harris.

